# Relationship of Wine Neophobia Levels with Demographic Factors and Wine Consumption Behavior in Spanish Consumers

**DOI:** 10.3390/nu17040687

**Published:** 2025-02-14

**Authors:** Celia Criado, Maria Ángeles Pozo-Bayón, Laura Domínguez, Virginia Fernández-Ruiz, Carolina Muñoz-González

**Affiliations:** 1 Instituto de Investigación en Ciencias de la Alimentación, CSIC-UAM, C/ Nicolás Cabrera, 9, Fuencarral-El Pardo, E-28049 Madrid, Spain; celia.criado@csic.es (C.C.); m.delpozo@csic.es (M.Á.P.-B.); c.munoz@csic.es (C.M.-G.); 2 Nutrition and Food Science Department, Pharmacy Faculty, Complutense University of Madrid (UCM), Plaza Ramón y Cajal, s/n, E-28040 Madrid, Spain; ladoming@ucm.es

**Keywords:** food neophobia, wine neophobia, consumer behavior, demographic factors, willingness to pay

## Abstract

Background/Objectives: Wine neophobia identifies segments of consumers who are reluctant to consume new or unfamiliar wines. This study examined the wine neophobia levels of a cohort of 376 Spanish wine consumers and the differences in demographics, wine consumption, and food neophobia according to their degree of wine neophobia. To that end, a specific survey with demographic data, wine consumption habits, and neophobia levels was designed and administered to Spanish consumers. Methods: The Wine Neophobia Scale (WNS) and Food Neophobia Scale (FNS) were used, and data collected were statistically analyzed (chi-square test, Pearson correlation analyses, and principal component analysis (PCA)). Two different clusters were identified: high- and low-wine-neophobic groups (HWN and LWN, respectively). Results: Results indicated significant differences in gender, marital status, and employment between groups. The HWN group was generally formed by women and singles, whereas participants with a partner (not married) and employed individuals were mostly in the LWN group. Overall, HWN consumers were characterized by consuming wine less frequently, preferring fruity and “softer” wines (e.g., whites and sparkling wines) or wine mixed with soda, and being willing to pay less money (“less than 5 €”) to buy wine on a daily basis than low-neophobics, who preferred red reserve wines with higher sourness and astringency and were willing to pay for more expensive wines. Finally, a direct relation has been observed between wine and food neophobia, as the LNW group reported lower scores on items relative to greater openness to the consumption of new foods. Conclusions: The present study provides for the first time insights into the relationship between wine neophobia, demographics, and wine consumption behavior in Spanish consumers, which can be useful to the wine industry for the development of personalized wines. This approach can aid wine market segmentation as well as product innovation.

## 1. Introduction

Neophobia is the persistent and abnormal fear of anything new. In the context of food, neophobia is defined as an unwillingness to eat new or unfamiliar foods contributing to personal eating behavior traits that influence the choice and sensory acceptance of new foods [1,2]. Different factors might contribute to the degree of food neophobia, from sensory aversions towards specific characteristics of the food product in question (texture, smell, taste, appearance, etc.) to the relationship with a previous negative experience, or the fear of negative consequences that can occur following the consumption of specific food products [3].

According to scientific evidence, food neophobia negatively impacts consumers’ health, as those individuals with a high degree of food neophobia tend to follow a less varied diet, consuming the same type of food items repeatedly. In addition, some studies have suggested an indirect relationship between food neophobia and consumption of food of plant origin like fruits and vegetables [4,5]. To measure this psychological behavior, Pliner et al. [2] developed a validated tool called the Food Neophobia Scale (FNS). However, as food neophobia differs depending on the product tested [6], different scales must be developed to study specific products, such as wine.

In the case of wine, Ristic et al. [7] adapted the FNS to wine, obtaining the Wine Neophobia Scale (WNS). This scale allows us to measure the reluctance of consumers to try novel wines, being one powerful tool for the development of product innovations (e.g., natural wines, extract additions, changes in the production process, etc.) for the wine industry. Considering that consumer perception has a significant impact on the adoption and success of new wines commercialized in the market [8], reluctance to try novel wines constitutes an important obstacle to embracing these innovations, and a better understanding of wine neophobia is needed to provide guidance to the industry and allow for the development of optimal marketing strategies. Moreover, since neophobia might vary depending on a great number of factors related to the product itself as well as individual characteristics (age, sex, income, level of education, etc.) [9,10], all these factors need to be considered to develop new wines. Scarce scientific studies about wine neophobia have been published in the literature. To the authors’ knowledge and based on a bibliographic review performed in this study, only a few authors such as Zhu et al. (2023), Pickering et al. (2021), Nguyen et al. (2019), Nieto-Villegas et al. (2022), Rabadán and Bernabéu (2021a,b), and Rabadán (2021) have investigated and evaluated the level of wine neophobia and its influence in consumers’ decision-making.

Zhu et al. (2023) reported that wine sensory attributes, such as aroma, visual appearance, mouthfeel, and taste, are important factors for consumer acceptance of these products and that they influence each other [11]. As an example, certain visual characteristics of a wine may have an effect on how its aroma is perceived by consumers and, at the same time, its aroma itself has an essential role in shaping the overall flavor experience. Regarding individual characteristics, to the authors’ knowledge, wine neophobia has been scarcely studied. Recently, Pickering et al. used a modified WNS to investigate demographic and behavioral correlations of wine neophobia in Canadian consumers [10]. These authors claimed that neophobes had lower household income, education, and wine involvement and reported consuming fewer wine styles than neophiles. However, these findings could vary depending on the population studied. Regarding other studies assessing the level of wine neophobia, Nguyen et al. (2019) performed a cross-cultural examination of Australian, Chinese, and Vietnamese consumers’ attitudes towards a new wine containing extracts of a specific woody mushroom which was used in traditional Chinese medicine. These authors revealed that most of the recruited consumers would accept the new wine; however, differences across cultures were found, as those consumers from Australia and China were significantly less neophobic than the Vietnamese participants [12]. In the particular case of Spanish consumers, food neophobia and wine neophobia have recently been investigated by Nieto-Villegas et al. (2022), Rabadán and Bernabéu (2021a,b), and Rabadán (2021) [13,14,15,16]. In these studies, the relationship of neophobia with new consumption trends for organic and sustainable wines has been investigated; however, only the relationship between neophobia and organic wines has been assessed, and no other factors that directly affect consumers, such as the preference for other types of wines, e.g., sparkling wines.

Apart from these references, and although Spain is one of the three largest wine producers, to the authors’ knowledge, no scientific evidence about the potential influence of demographics and consumption behavior on the wine neophobia levels in Spanish consumers is available. To address this existing gap, the present study aimed to characterize Spanish consumers’ reluctance towards novel wines by examining and analyzing for the first time the differences in demographics (age, gender, education level, marital status, employment, monthly food expenditure, usual place of residence), wine consumption habits (wine frequency consumption, wine preferences, willingness to spend on a bottle of wine, preference for protected designation of origin (PDO)), and food neophobia level according to participants’ degree of wine neophobia (high and low) through an online survey. As a first approach, it is hypothesized that wine neophobia levels are significantly influenced by socio-demographic characteristics and wine drinking behavior, and that the higher the level of food neophobia, the greater the neophobic behavior towards wine. It is expected that the findings of the present study provide relevant information to understand the drivers of wine behavior and provide guidance to the wine industry for product innovation.

## 2. Materials and Methods

### 2.1. Survey

An online survey was designed and administered to Spanish consumers by social media from March to May 2021. This survey was divided into 4 sections: (1) demographic information, (2) wine consumption behavior, (3) wine neophobia, and (4) food neophobia. Questions for Sections 1 and 2 (demographics and wine consumption behavior) were specifically designed by the authors of the present work, whereas items related to wine neophobia (Section 3) and food neophobia levels (Section 4) were the official ones included in the Wine Neophobia Scale (WNS) and Food Neophobia Scale (FNS), which have already been validated in the Spanish language by Ristic et al. (2016) and Fernández-Ruiz et al. (2013), respectively. The details for each of the sections are described below.

This complete questionnaire was officially approved by CSIC Ethics Committee (008/2021, 26 February 2021). Regarding the sampling procedure, random sampling was performed to gather a representative sample from a population in which each individual has an equal possibility of being selected in the present study. Inclusion criteria called for people over 18 years of age and regular wine consumers (at least once a month). Out of 828 consumers who were approached, a total of 564 individuals reported to have drunk wine at least once in the previous month, and they were selected to be interviewed. In terms of exclusion criteria, individuals below 18 years as well as those who did not complete the designed survey were discarded, so a total of 376 consumers were finally considered in the study.

#### 2.1.1. Section 1: Demographic Information

In the first section of the questionnaire, respondents were asked to provide their age, gender, educational level, marital status, employment, monthly food expenditure, and usual place of residence. Age groups were divided into 18–40 and over 40 years old. The educational level of the individuals was divided into no schooling, primary education, secondary education, upper secondary education, and bachelor’s or master’s degree (or equivalent). Marital status information was grouped in 5 categories as follows: married, unmarried partner, divorced, single, and widowed. Employment was categorized as homemaker, self-employed, unemployed, employed, student, and retired. The approximate monthly food expenses were divided into less than 200 €, 201–400 €, 401–600 €, and more than 600 €. Finally, information regarding participants’ usual place of residence was clustered in 6 groups: living with friends or flatmates, living with relatives (parents, grandparents, etc.), living with a partner, living with a partner and/or children, living alone, and university residence.

#### 2.1.2. Section 2: Wine Consumption Behavior

Section 2 consisted of six questions. The first one concerned wine consumption frequency. Respondents had to rate how often they consume wine, choosing between 3 options: daily/weekly, only on weekends, and occasionally. Then, they were asked about what kind of wine they prefer to drink (white wine, rosé wine, young red wine, reserve red wine, and sparkling wine). Then, there were two questions to learn how much people would be willing to spend on a bottle of wine (750 mL), one about the expenditure on a bottle for everyday consumption, and another question about the price for a bottle to be drunk on a special occasion. The price options were less than 5 €, from 5 to 10 €, from 10 to 20 €, and more than 20 €. Finally, respondents were asked if wine was their preferred alcoholic beverage and whether they had a preference for a protected designation of origin (PDO).

#### 2.1.3. Section 3: Wine Neophobia (WN)

To measure WN, the Wine Neophobia Scale (WNS) developed by Ristic et al. [17] was adapted and used (Table 1).

Wine consumers evaluated each item using a 7-point Likert scale (1 = “strongly disagree” to 7 = “strongly agree”). Scores were summed across the 7 questions (correcting for reverse-keyed items) for a total score out of 49. Higher scores were indicative of higher wine-neophobic levels. Reliability of the WNS was assessed by calculating internal consistency with Cronbach’s alpha and intra-class correlation.

#### 2.1.4. Section 4: Food Neophobia (FN)

Food neophobia levels were assessed through the Food Neophobia Scale (FNS), a psychometric tool which provides with high precision a standardized measure of consumers’ reluctance to try new foods [2]. Considering that participants of this study were Spanish consumers, an adapted version of the FNS translated to the Spanish language and previously validated by Fernández-Ruiz et al. was used [18]. Participants responded in a self-applied way that evaluated their degree of food neophobia using a 7-point Likert scale from “strongly disagree” (1 point) to “strongly agree” (7 points) (Table 2). Scale items were composited to form a trait-level food neophobia score. Two of the ten items of the original version of the FNS were discarded in this questionnaire, as different authors recommended this in the scientific literature [18,19,20]. Scores were summed across the 8 questions (correcting for reverse-keyed items) for a total score out of 56. Higher scores were indicative of higher food-neophobic levels. Once again, Cronbach’s alpha was calculated to evaluate the internal consistency of the scale.

### 2.2. Data Analysis

The data collected were analyzed using XLSTAT (version 2022.2.1.1262, Addinsoft, New York, NY, USA). An analysis of Cronbach’s alpha was conducted to determine whether the results obtained using the Wine Neophobia Scale and Food Neophobia Scale (WNS and FNS) were reliable. By calculating this parameter, the authors could compare the amount of shared variance among the items with the amount of overall variance. For comparison of categorical data, a chi square test was used to address whether a relationship existed between two variables. Additionally, correlations between WNS and FNS results were analyzed using Pearson correlation analyses measuring the strength of the linear relationship between two variables. Finally, data obtained were evaluated by examining the factor structure of the FNS scores by means of principal component analysis (PCA). All analyses were conducted at a significance level of 5%.

## 3. Results

### 3.1. Wine Neophobia

**Figure 1:**
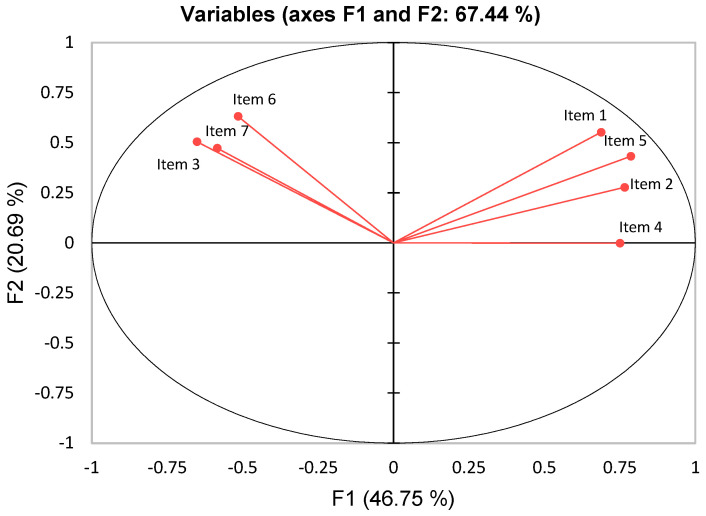
Correlation coefficients of items of the WNS with the first two principal components.

Wine Neophobia Scores were calculated, and a total of three groups were formed based on their levels of wine neophobia: a high-wine-neophobic group (mean = 25.62, SD = 2.70), medium-wine-neophobic group (mean = 16.67, SD = 1.96), and low-wine-neophobic group (mean = 9.72, SD = 2.22). Only the two extreme groups were considered for further data comparison: the high-wine-neophobic group (HN, n = 188) and the low-wine-neophobic group (LN, n = 188). The reliability of the adapted and validated Spanish-language version of the WNS used in this work was assessed by calculating internal consistency with Cronbach’s alpha = 0.720. Data obtained were assessed through principal component analysis (PCA) to investigate the construct validity of the WNS. As shown in Figure 1, 46.75% and 20.69% of the variance was explained by first and second principal components (F1 and F2), respectively. Items 1, 2, 4, and 5 were located in the first quadrant of the graph and showed high and positive correlation to F1, whereas items 3, 6, and 7 were located in the second quadrant and were correlated with F2. The PCA results clearly separate in different quadrants of the graph the reversed items from the unreversed ones (positive correlation). Thus, the Spanish version of the WNS performs properly with Spanish consumers recruited in this study.

#### 3.1.1. Differences in Demographic Data Between Wine-Neophobic Groups

Table 3 shows the differences in demographic data between the high- and low-wine-neophobic groups. As can be seen, no significant differences were observed in the age, education level, monthly expenditure on food, and household data of the respondents between the two wine-neophobic groups.

Significant differences between the wine-neophobic groups were observed for gender (*p* < 0.001), marital status (unmarried partner and single) (*p* < 0.013 and *p* < 0.028, respectively), and employment (employed) (*p* < 0.021). Thus, the high-wine-neophobic group presented a significantly higher number of females and single respondents, while the low-wine-neophobic group presented a high number of respondents who had partners but were unmarried and employed people. Regarding educational level, most respondents had a bachelor’s or master’s degree. Interestingly, although no significant differences were observed between the two groups, a tendency (*p* = 0.066) was observed for those with bachelor’s or master’s degrees, a higher number of which were in the low-wine-neophobic group.

#### 3.1.2. Differences in Wine Consumer Behavior Data Between Wine-Neophobic Groups

##### Frequency of Wine Consumption

**Figure 2:**
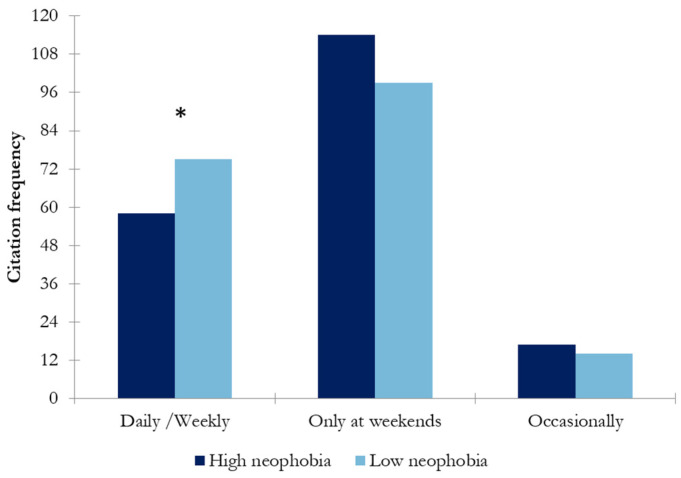
Results of chi-square analysis showing the frequency of wine consumption in both neophobia groups (high wine neophobia and low wine neophobia) (the asterisk indicates significant differences (*p* < 0.05) between wine-neophobic groups).

As can be seen in Figure 2, most respondents consume wine at the weekend or on a daily/weekly basis. Only a small percentage of respondents consume wine occasionally. Significant differences were observed between wine-neophobic groups only in daily/weekly (*p* < 0.047) frequency of wine consumption. The daily/weekly consumption of the low-wine-neophobic group was 23% higher than that of the high-wine-neophobic group. However, no significant differences were observed between wine-neophobic groups in respondents that consume wine only at weekends or occasionally. This could indicate that among wine consumers, wine neophobia could be an important factor in wine consumption, but only for high-frequency consumers.

##### Wine Preference

**Figure 3:**
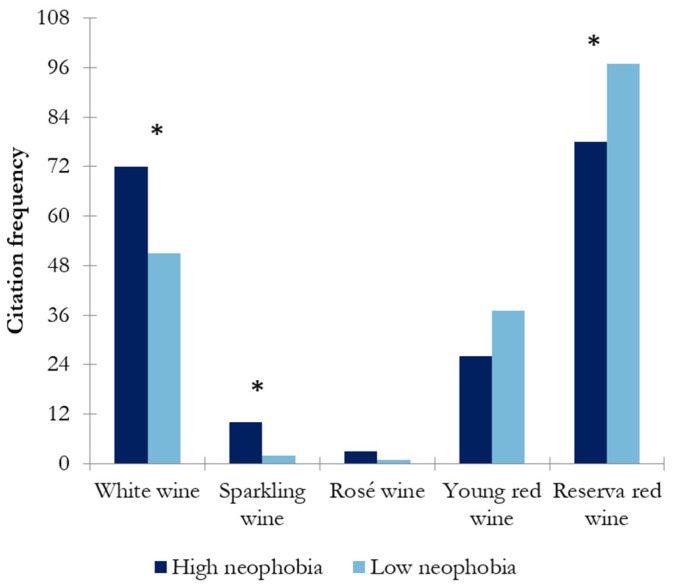
Results of chi-square analysis showing the wine preference in both wine-neophobic groups (the asterisk indicates significant differences (*p* < 0.05) between wine-neophobic groups).

As can be seen in Figure 3, reserve red wine and white wine were the most preferred wines among Spanish wine consumers, sparkling and rosé wine the least. Analyzing the wine preference between wine-neophobic groups (Figure 2), significant differences (*p* < 0.05) were observed for white wine, sparkling wine, and reserve red wine. The high-wine-neophobic group significantly preferred the white wine and sparkling wine over the reserve red wine.

##### Everyday Spending on Wine

**Figure 4:**
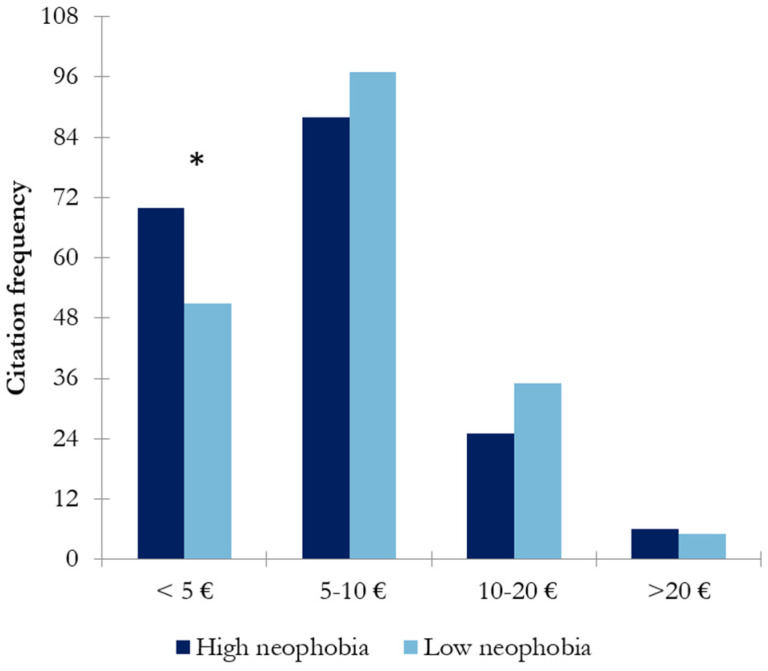
Results of chi-square analysis showing the intended expenditure on a bottle for daily consumption in both wine-neophobic groups (the asterisk indicates significant differences (*p* < 0.05) between neophobic groups).

Regarding the willingness to spend more or less money on a bottle of wine on a daily basis, the highest scores were observed for the less expensive options, those costing 5–10 € and <5 euros (Figure 4).

Significant differences between wine-neophobic groups were only observed in the case of the lowest price, <5 €. In this case, a difference of 16% was observed between groups, with the high-wine-neophobic group presenting a higher disposition toward acquiring these types of products.

##### Spending Money on Wine When Celebrating a Special Occasion

**Figure 5:**
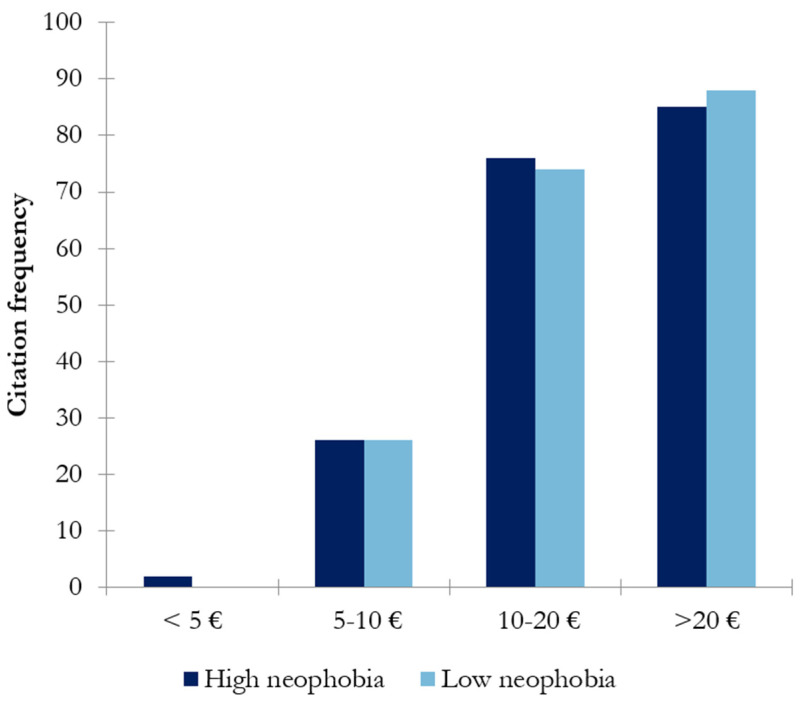
Results of chi-square analysis showing the intended expenditure on a bottle for celebrating a special occasion in both wine-neophobic groups.

Regarding the intention to spend money on a bottle of wine for a special occasion (Figure 5), the highest scores were observed for the most expensive options, those costing 10–20 € and >20 €. However, no differences between the wine-neophobic groups were observed.

##### PDO (Protected Designation of Origin) Wine Preference

**Figure 6:**
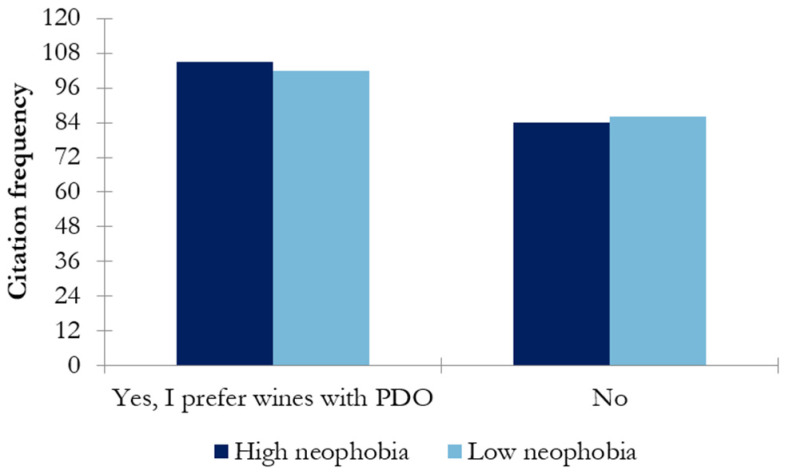
Results of chi-square analysis showing the preference for a PDO wine in the choice of wine in both wine-neophobic groups.

According to the results obtained in this study, no significant differences in PDO wine preference were observed between the high- and low-wine-neophobic groups (Figure 6).

##### Preference of Wine over Other Alcoholic Beverages

**Figure 7:**
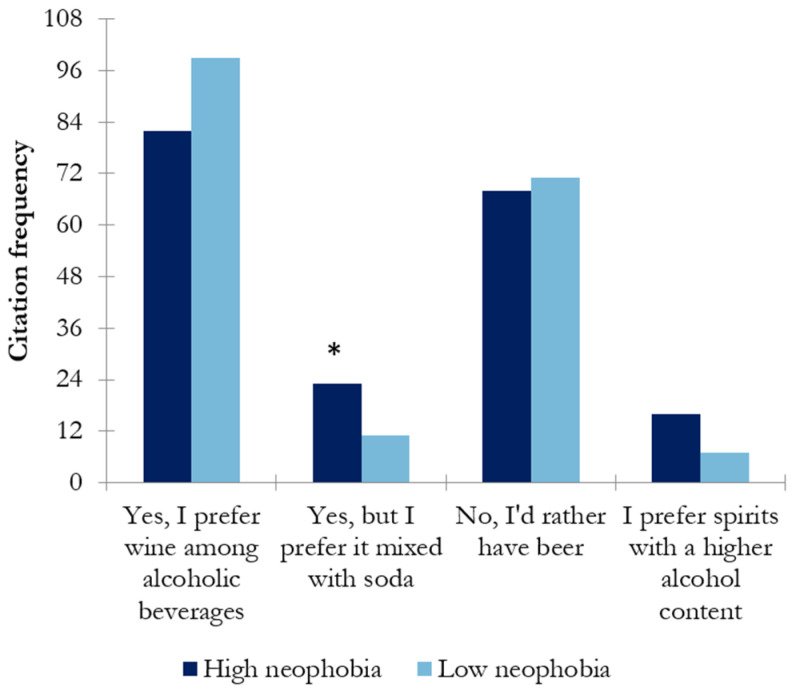
Results of chi-square analysis showing the main choice during the consumption of alcoholic beverages in both wine-neophobic groups (the asterisk indicates significant differences (*p* < 0.05) between neophobic groups).

The preference of wine over other alcoholic beverages was analyzed, as shown in Figure 7. Most responses indicated that wine was the beverage of choice for respondents when consuming alcoholic beverages, followed by beer. However, a small percentage of Spanish participants in this study preferred to drink wine mixed with soda. In this case, a significant difference was observed between wine-neophobic groups, with the latter being an option more frequently chosen by the high-wine-neophobic group.

#### 3.1.3. Differences in Food Neophobia Between Wine-Neophobic Groups

The mean value of food neophobia in individuals recruited in this work was 20.52 (SD 8.84). A Cronbach’s alpha = 0.81 was obtained, confirming the reliability of the results.

**Figure 8:**
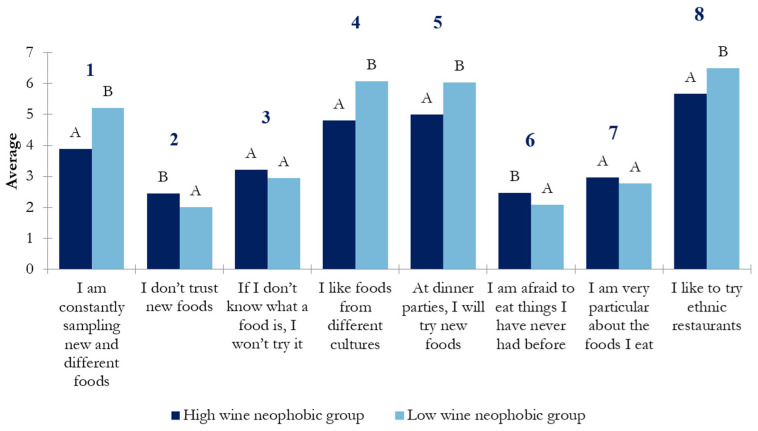
*t*-test analysis of the analyzed FNS items between wine-neophobic groups. Letters A and B indicate significant differences (*p* < 0.05) between wine-neophobic groups. The numbers 1 to 8 indicate the corresponding item.

Differences in food neophobia levels between the wine-neophobic groups are represented in Figure 8. As can be seen, significant differences between wine-neophobic groups were observed for items 1 (“I am constantly sampling new and different foods”), 2 (“I don’t trust new foods”), 4 (“I like foods from different cultures”), 5 (“At dinner parties, I will try new foods”), 6 (“I am afraid to eat thing I have never had before”), and 8 (“I like to try ethnic restaurants”). The low-wine-neophobic group scored higher for all the positive items related to food consumption (items 1, 4, 5, and 8) than the high-wine-neophobic group. Conversely, the high-wine-neophobic group scored significantly higher than the low-wine-neophobic group on two of the items related to reluctance to try new food (items 2 and 6).

## 4. Discussion

To the authors’ knowledge, the present study provides for the first time insights into the differences in demographics, wine consumption habits, and food neophobia levels between high and low wine neophobia in Spanish consumers. This approach can aid with wine market segmentation and product innovation.

Regarding demographic data, no differences of age between the two wine-neophobic groups were observed (Table 3), which agrees with previous findings published by other authors in the scientific literature [10,12]. In contrast, Ristic et al. [7] found that wine neophobes tend to be older than wine neophiles, while Demattè et al. (2014), Fernández-Ruiz et al. (2013), Lähteenmäki and Arvola (2001), Schnettler et al. (2013), Tuorila and Cardello (2001), and Tuorila et al. (2001) reported that older adults were less neophobic [18,21,22,23,24,25].

Divergences across studies could be explained by differences in the studied population. Accordingly, there were no significant differences in education, food expenditure, or household situation between the high-wine-neophobia and low-wine-neophobia groups in this study (Table 3). Nevertheless, it is important to mention that most of the participants in the present work had a high level of education (bachelor’s and master’s degree), in connection with a tendency (*p* = 0.066) observed in the wine-neophobic groups. That is, the low-wine-neophobic group presented a higher number of participants with bachelor’s and master’s degrees than the high-wine-neophobic group. This finding agrees with the fact that wine neophobes have been reported as less educated in other studies [7,10,13]. The same tendency has been observed in the scientific literature regarding the degree of food neophobia. The higher the level of education, the lower the level of food neophobia [25,26,27,28,29].

In the present study, the high- and the low-wine-neophobic groups were significantly different in relation to gender, being single or having a partner without being married, and being employed (Table 3). In this respect, the high-wine-neophobic group presented a high number of women, which confirms previous findings from another group of research in Spanish consumers. The reason behind this finding was previously attributed to the fact that women are generally less interested in wine [13]. The high-wine-neophobic group also presented a higher percentage of single people, and a low number of individuals having a partner without being married. All these findings require further exploration in future studies. Finally, the low-wine-neophobic group presented a higher number of employed people, which indirectly could be related to their level of income.

Wine neophobia might be a factor influencing wine consumption behavior. In this study, the differences between high- and low-wine-neophobic groups regarding the frequency of consumption were explored. The results showed that the low-wine-neophobic group scored significantly higher in daily/weekly consumption than the high-wine-neophobic group (*p* < 0.047) (Figure 1). That is, wine neophiles drink more frequently than neophobes, in accordance with previous findings [10]. Regarding preference among different types of wines, the high-wine-neophobic group had a greater preference for white wines and sparkling wines than the low-wine-neophobic group, who preferred red wines (reserve red wines) (Figure 2). A possible reason behind these results is the combination of aromatic properties and mouthfeel complexity of the wines. These results suggest that wine-neophobic consumers prefer those that are considered to be sweeter or softer than red wines, which have higher sourness and astringency. Accordingly, other studies of Spanish wine consumers have observed that low-wine-neophobia individuals are more likely to choose red and aged wines [14,15,30] and that a liking for red wines remarkably increased according to age in the high-neophobic group [31]. In addition to the type of wine, another known source of variation in choosing a wine is its price. In this study, Spanish consumers were asked about their willingness to buy wine in two situations: on a daily basis (Figure 3) or for a special occasion (Figure 4). To the authors’ knowledge, no study has examined how willingness to pay varies among neophobic groups depending on the occasion (e.g., daily and special occasions). For daily-basis consumption, there were significantly more neophobes than neophiles in the “less than 5 euros” category. This may suggest that neophobes are likely to pay less per bottle of wine as compared to neophiles, in agreement with previous findings [10]. This finding, coupled with the relationship between neophobia and employment reported above, may support the idea that neophobes are less likely to spend limited resources on novel products. However, no significant differences were observed between wine-neophobic groups in terms of the price participants were willing to pay to buy a wine for a special occasion (Figure 4).

It is estimated that 51.4% of the wine produced in Spain is PDO wine, compared to 41.5% non-PDO or PGI wine (Protected Geographical Indication) [32]. For that reason, the preference of neophobes for wines from a PDO was investigated in this study (Figure 5). Surprisingly, no significant differences between neophobic groups were observed regarding PDO wine. New studies investigating specific PDOs should be conducted in the future.

The preference of Spanish consumers for a specific alcoholic drink was investigated according to their wine neophobia levels (Figure 6). The only significant difference observed between wine-neophobic groups was for wine mixed with soda, with higher values given by the high-wine-neophobics. This could be related to the fact that the sensory characteristics of the wine/soda mixture could be more familiar and softer to them, and thus accepted.

Regarding food neophobia degree, the FN mean score obtained (20.52 ± 8.84) indicated that Spanish consumers who participated in this study had a very low neophobic level in comparison with other individuals from Australia (32.9 ± 8.9) [7], Asia (32.3 ± 16.97) [33], Finland (31.2 ± 11.5) [34], and the Netherlands (30.1 ± 9.5) [35]. Only Norwegian and Chilean consumers reported lower levels of food neophobia (16.1 ± 7.3 and 17.5 ± 6.6, respectively) [23,36]. The relation of food neophobia with wine neophobia was also investigated in the present study (Figure 7). Results showed that the low-wine-neophobic group reported lower scores on the items from the FNS relative to greater openness to the consumption of new food. Conversely, the high-neophobic-group scored higher on the items of the FNS related to greater reluctance to consume novel food. This result highlights the relationship between the two scales and the consistency of the present results in agreement with previous findings.

## 5. Limitations and Future Research Directions

The present work has some limitations. On the one hand, this study did not assess the potential influence of the socio-economic status of participants on the levels of wine neophobia. This factor could have been interesting to consider in this work, as other authors have suggested in the scientific literature that lower household incomes were generally associated with high levels of wine neophobia. In this study, questions related to the socio-economic status were not included in the survey/questionnaire designed. In addition, questions related to general health interest, daily diet and food patterns, adherence to a varied and balanced diet like the Mediterranean Diet, as well as food restrictions of participants could have enriched the interpretation and discussion of results. On the other hand, wine preferences and attitudes toward unfamiliar wines are strongly influenced by cultural norms and regional wine traditions. This key point should be taken into consideration when comparing results obtained in other studies with participants from other countries and/or regions with different wine consumption habits or levels of exposure to wine diversity. All these factors could provide relevant information and would be of great interest for consideration in future research. Future research directions could focus on performing cross-cultural studies as well as examining the potential changes in wine neophobia levels over time (e.g., how exposure to diverse wines or changes in cultural trends might influence attitudes), as current studies are cross-sectional works that analyze participants’ attitudes at a single point in time.

## 6. Conclusions

Despite Spain being a country with a long tradition of wine consumption, wine neophobia has been scarcely explored. In the present work, the authors examined the differences between high- and low-wine-neophobic groups in terms of demographics, wine consumption behavior, and food neophobia. Results indicated significant differences in gender, marital status, and employment between wine-neophobic groups. Thus, the high-wine-neophobic group was formed by a higher number of women and singles, while the low-wine-neophobic group by a higher number of people with partners (without being married) and employed individuals. Although not to a significant degree, the low-wine-neophobic group was formed by a higher number of people with a high educational level. Moreover, authors found that wine neophobia levels are related to wine drinking behavior. In fact, wine consumption frequency and wine type preference were determined by the neophobic level, as neophobic participants reported consuming wine less frequently and preferred the fruity and “softer” wines like white and sparkling wines, while red reserve wines were preferred by the low-neophobic group. No significant differences between wine-neophobic groups were observed in terms of preference for PDO wine. However, significant differences were observed in the willingness to choose a wine according to its price, with the high-wine-neophobic group showing a greater preference for spending “less than 5 €” to buy wine for daily-basis consumption than the low-wine-neophobics. This might indicate that neophobes are likely to pay less per bottle of wine as compared to neophiles. Finally, a direct relation has been observed between wine and food neophobia.

Overall, the results obtained in this study suggest a relation between wine neophobia and demographics and consumption behavior that could be of great interest for the wine industry in the development of personalized wine strategies. The wine neophobia results obtained in the present study can be useful for targeted marketing strategies by identifying consumer behaviors, preferences, and hesitations across demographic factors. Understanding these nuances enables the wine industry and companies to design tailored strategies to overcome reluctant attitudes, reduce perceived risks, increase familiarity, and encourage consumers to explore and enjoy a wider range of wines. For instance, strategies directed to individuals with high levels of wine neophobia could be focused on emphasizing familiarity and reassurance by using well-known wine varieties and regions or trusted certifications, while wine products directed toward neophilic groups could highlight novelty and exclusivity, such as unique grape varietals or niche wine regions.

## Figures and Tables

**Table 1:** Items of the Wine Neophobia Scale (WNS) in English and Spanish languages.

Item	Statements in English	Statements in Spanish
1	I like going to places serving wines from different countries (R)	Me gusta ir a lugares donde sirven vinos de diferentes países (R)
2	I will drink almost any wine (R)	Bebería casi cualquier vino (R)
3	I am afraid to drink wines I have never had before	Me da miedo beber vinos que no he probado nunca
4	At social gatherings, I will try a new wine (R)	En celebraciones sociales, probaría cualquier vino (R)
5	I like wines from different countries (R)	Me gusta probar vinos de diferentes países (R)
6	If I do not know what wine it is, I won’t try it	Si no sé qué vino es, no lo pruebo
7	I do not trust new wines	No confío en vinos nuevos

(R) indicates negatively worded items for which scores were reversed for calculation of the WNS score.

**Table 2:** Items of the Food Neophobia Scale (FNS) in Spanish and English languages.

Items	Statements in English	Statements in Spanish
1	I am constantly sampling new and different foods (R)	Estoy constantemente probando alimentos nuevos y diferentes (R)
2	I don’t trust new foods	No confío en los alimentos nuevos
3	If I don’t know what a food is, I won’t try it	Si no conozco qué hay en un alimento, no lo pruebo
4	I like foods from different cultures (R)	Me gustan las comidas de países diferentes (R)
5	At dinner parties, I will try new foods (R)	En fiestas con comida, pruebo nuevos alimentos (R)
6	I am afraid to eat things I have never had before	Me da miedo probar alimentos que nunca he probado antes
7	I am very particular about the foods I eat	Soy muy especial con los alimentos que como
8	I like to try ethnic restaurants (R)	Me gusta probar nuevos restaurantes étnicos (R)

(R) indicates negatively worded items for which scores were reversed for calculation of the FNS score.

**Table 3:** Results of chi-square analysis of demographic data between the wine-neophobic groups.

Variables	Options	High-Wine-Neophobic Group	Low-Wine-Neophobic Group	Chi-Square (*p*)
Age	18–40 y.o.	92	101	0.301
	Over 40 y.o.	96	87	0.301
Gender	Female	124	92	0.001
	Male	64	96	0.001
Education level	No schooling	0	0	-
	Primary education	3	1	0.623
	Secondary education	11	3	0.053
	Upper secondary education	47	40	0.463
	Bachelor or master (or equivalent)	127	144	0.066
Marital status	Married	86	86	0.999
	Unmarried partner	31	52	0.013
	Divorced	7	7	1.000
	Single	62	42	0.028
	Widower	2	1	0.999
Employment	Homemaker	0	0	-
	Self-employment	16	15	0.999
	Unemployment	16	10	0.309
	Employed	99	122	0.021
	Student	40	33	0.434
	Retired	17	8	0.096
Monthly expenditure on food	<200 euros	83	82	0.999
	201–400 euros	43	51	0.405
	401–600 euros	34	32	0.892
	>600 euros	28	23	0.547
Household	Living with friends or flatmates	18	17	0.999
	Living with relatives (parents, grandparents, etc.)	42	31	0.192
	Living with my partner	51	46	0.637
	Living with my partner or my kids	61	79	0.070
	I live alone	16	14	0.849
	Universitary residence	0	1	0.999

## Data Availability

The original contributions presented in this study are included in the article. Further inquiries can be directed to the corresponding author.

## References

[1] Capiola A., Raudenbush B. (2012). The Effects of Food Neophobia and Food Neophilia on Diet and Metabolic Processing. Food Nutr. Sci..

[2] Pliner P., Hobden K. (1992). Development of a scale to measure the trait of food neophobia in humans. Appetite.

[3] Del Campo C., Bouzas C., Monserrat-Mesquida M., Tur J.A. (2023). Assessing Food Preferences and Neophobias Among Spanish Adolescents from Castilla–La Mancha. Foods.

[4] Hazley D., McCarthy S.N., Stack M., Walton J., McNulty B.A., Flynn A., Kearney J.M. (2022). Food neophobia and its relationship with dietary variety and quality in Irish adults: Findings from a national cross-sectional study. Appetite.

[5] Knaapila A.J., Sandell M.A., Vaarno J., Hoppu U., Puolimatka T., Kaljonen A., Lagström H. (2015). Food neophobia associates with lower dietary quality and higher BMI in Finnish adults. Public Health Nutr..

[6] Van Trijp H.C.M. (1994). Product-related Determinants of Variety-Seeking Behavior for Foods. Appetite.

[7] Ristic R., Johnson T.E., Meiselman H.L., Hoek A.C., Bastian S.E.P. (2016). Towards development of a Wine Neophobia Scale (WNS): Measuring consumer wine neophobia using an adaptation of the Food Neophobia Scale (FNS). Food Qual. Prefer..

[8] Barisan L., Galletto L., Franceschi D., Caracciolo F. (2024). From vineyard to glass: Measuring consumers’ willingness to pay for innovative rootstock-produced wine through an experimental auction. J. Clean. Prod..

[9] Białek-Dratwa A., Staśkiewicz-Bartecka W., Kiciak A., Wardyniec A., Grajek M., Aktaç Ş., Çelik Z.M., Sabuncular G., İslamoğlu A.H., Kowalski O. (2024). Food Neophobia and Avoidant/Restrictive Food Intake Among Adults and Related Factors. Nutrients.

[10] Pickering G.J., Dale G., Kemp B. (2021). Optimization and application of the wine neophobia scale. Beverages.

[11] Zhu Y., Su Q., Jiao J., Kelanne N., Kortesniemi M., Xu X., Zhu B., Laaksonen O. (2023). Exploring the Sensory Properties and Preferences of Fruit Wines Based on an Online Survey and Partial Projective Mapping. Foods.

[12] Nguyen A.N., Johnson T.E., Jeffery D.W., Danner L., Bastian S.E.P. (2019). A cross-cultural examination of Australian, Chinese and Vietnamese consumers’ attitudes towards a new Australian wine product containing Ganoderma lucidum extract. Food Res. Int..

[13] Nieto-Villegas R., Rabadán A., Bernabéu R. (2022). A gender approach to wine innovation and organic wine preferences. Cienc. Tec. Vitivinic..

[14] Rabadán A., Bernabéu R. (2021). An approach to eco-innovation in wine production from a consumer’s perspective. J. Clean. Prod..

[15] Rabadán A., Bernabéu R. (2021). A systematic review of studies using the Food Neophobia Scale: Conclusions from thirty years of studies. Food Qual. Prefer..

[16] Rabadán A. (2021). Consumer attitudes towards technological innovation in a traditional food product: The case of wine. Foods.

[17] Ristic R., Danner L., Johnson T.E., Meiselman H.L., Hoek A.C., Jiranek V., Bastian S.E.P. (2019). Wine-related aromas for different seasons and occasions: Hedonic and emotional responses of wine consumers from Australia, UK and USA. Food Qual. Prefer..

[18] Fernández-Ruiz V., Claret A., Chaya C. (2013). Testing a Spanish-Version of the Food Neophobia Scale. Food Qual. Prefer..

[19] Domínguez L., Fernández-Ruiz V., Sánchez-Mata M.C., Cámara M. (2019). Food neophobia: Spanish case study related to new formulations based on traditional ‘gazpacho’. Acta Hortic..

[20] Ritchey P.N., Frank R.A., Hursti U.K., Tuorila H. (2003). Validation and cross-national comparison of the food neophobia scale (FNS) using confirmatory factor analysis. Appetite.

[21] Demattè M.L., Endrizzi I., Gasperi F. (2014). Food neophobia and its relation with olfaction. Front. Psychol..

[22] Lähteenmäki L., Arvola A., Frewer L.J., Risvik E., Schifferstein H. (2001). Food neophobia and variety seeking—Consumer ear or demand for new food products. Food, People and Society.

[23] Schnettler B., Crisóstomo G., Sepúlveda J., Mora M., Lobos G., Miranda H., Grunert K.G. (2013). Food neophobia, nanotechnology and satisfaction with life. Appetite.

[24] Tuorila H., Cardello A. (2001). Consumer responses to an off-flavor in juice in the presence of specific health claims. Food Qual. Prefer..

[25] Tuorila H., Lähteenmäki L., Pohjalainen L., Lotti L. (2001). Food neophobia among the Finns and related responses to familiar and unfamiliar foods. Food Qual. Prefer..

[26] D’Antuono L.F., Bignami C. (2012). Perception of typical Ukrainian foods among an Italian population. Food Qual. Prefer..

[27] Meiselman H.L., King S.C., Gillette M. (2010). The demographics of neophobia in a large commercial US sample. Food Qual. Prefer..

[28] Sanjuán-López A., Philippidis G., Resano-Ezcaray H. (2011). How useful is acceptability to explain economic value? An application on the introduction of innovative saffron products into commercial markets. Food Qual. Prefer..

[29] Schickenberg B., Van Assema P., Burg J., De Vries N. (2006). Level of food neophobia in Dutch adults and association with familiarity with and willingness to try new healthful food products. Appetite.

[30] Bernabéu R., Díaz M., Oliveira F. (2016). Consumer preferences for red wine in the Spanish market. Cienc. Tec. Vitivinic..

[31] Laureati M., Spinelli S., Monteleone E., Dinnella C., Prescott J., Cattaneo C., Pagliarini E. (2018). Associations between food neophobia and responsiveness to “warning” chemosensory sensations in food products in a large population sample. Food Qual. Prefer..

[32] Ministerio de Agricultura Pesca y Alimentación Informe del Consumo de Alimentación en España 2020 (Issue Nov 2021). https://www.mapa.gob.es/es/alimentacion/temas/consumo-tendencias/panel-de-consumo-alimentario/resumen-anual-de-la-alimentacion/.

[33] Edwards J.S.A., Hartwell H.L., Brown L. (2010). Changes in food neophobia and dietary habits of international students. J. Hum. Nutr. Diet..

[34] Knaapila A., Silventoinen K., Broms U., Rose R.J., Perola M., Kaprio J., Tuorila H.M. (2011). Food neophobia in young adults: Genetic architecture and relation to personality, pleasantness and use frequency of foods, and body mass index—A twin study. Behav. Genet..

[35] Schickenberg B., Van Assema P., Brug J., De Vries N.K. (2008). Are the Dutch acquainted with and willing to try healthful food products? The role of food neophobia. Public Health Nutr..

[36] Helland S.H., Bere E., Bjørnarå H.B., Øverby N.C. (2017). Food neophobia and its association with intake of fish and other selected foods in a Norwegian sample of toddlers: A cross-sectional study. Appetite.

